# Cancerous Affection of the Thalami Nervorum Opticorum

**Published:** 1812-07

**Authors:** J. S. Blount

**Affiliations:** Paris


*To the Editors of the Medical and Physical Journal.*

GENTLEMEN,

YOU were so good as to insert in one of the former Numbers of your Journal some communications which I sent you from Paris: I beg leave to add the following to them, and am, Gentlemen, Your's, &c.

*Paris*, 1812. J. S. BLOUNT.

*Two Cases of Gutta Serena, produced by Cancerous Affection of the Thalami nervorum opticorum.*

A girl, aged 21, of a robust constitution, silent and morose disposition, and who never had menstruated regularly, became subject to epilepsy without any known cause; she had remarked for some time previous that when she worked much at her needle her eyes became red and painful, headache likewise came on: each attack of epilepsy was followed by a state of drowsiness, during the continuance of which no weight upon the head could be supported; whatever was put thereon was immediately torn off. After about three months, these attacks became much more frequent; vision was gradually impaired, became weaker and weaker, and was at last quite lost: the patient then fell into a continual state of coma, the pulse was weak, slow, and depressed, respiration slow, the eyes closed, the eye-balls fixed, both the upper eyelids, but especially the left, appeared paralysed. In this state she was carried to the Hotel Dieu at Paris, where they found it necessary to prick her with pins in order to rouse her. When questioned about the seat of pain, she carried her hand to her head, and immediately fell into the same comatose state. Bleeding from the feet, the exhibition of an emetic, and blisters to the legs, restored her so far as to enable her to speak a few words; but, soon falling into the same lethargy, her face became discomposed, her tongue black, and, when she was supposed on the point of dying from debility, violent convulsions came on, in the middle of which she expired, five months after the first attack of epilepsy, and five weeks after the comatose state became continual. The vessels of the brain and cerebellum were much injected; several ounces of bloody serum were found in the lateral ventricles; the left thalamus of the optic nerve was quite schirrous; the right thalamus was larger than natural, grey, and hard externally, blackish and of a bacon-like consistence internally, having the appearance of true cancer; all that portion of brain immediately surrounding these parts was hard and schirrous.—*Journal de Medecine de Corvisart Leroux, etc. for Nov.* 1810, *communicated by Dr. Beauchêre.*

A hard-working man, of a good constitution, aged 32 years, entered the Hotel Dieu for a blindness with which he had

3 been been affected for some months, which had been preceded, and was accompanied, by continual head-ache. No amelioration had resulted from the various means which had been made use of, and, when he entered the hospital, the head-ache was general and unceasing, whatever might be the position of the head. The intellectual faculties were unimpaired, sleep natural, and the eyes to all appearance in a sound state, though the pupils were much dilated, and the iris scarcely affected by a strong light; all the other functions of the body appeared to be in a state of perfect health. No cause could be assigned for this complaint; general means to excite the nervous system were used, but without the least effect; and, three weeks after his admission, his eyes became insensible to the strongest light. His mental faculties remaining good, it was thought useless to torment him with remedies, they were therefore laid aside: in a few days after he fell into a state of coma, which continued for about twelve or fifteen hours, when he died. The vessels of the membranes of the brain were rather turgid; the upper circumvolutions of the brain a little effaced; each lateral ventricle contained about two ounces of a limpid serous fluid. The thalami nervorum opticorum were found in a cancerous state, and this affection comprised the two portions of the brain called the middle lobes; the membranes of the eye and the optic nerve were in their natural state, as was also every other part of the body. From this observation we may conclude, 1st, that Gutta Serena may depend on a schirrous or cancerous state of the middle lobes of the brain; 2d, that blindness in this case is only a symptom of another disease, which is both incurable and mortal; and lastly, that it is essential to find out the means of recognising the Gutta Serena dependant on this cause, not to torment patients with useless remedies.—*Journal de Medecine de Corvisart Leroux, etc. for Feb.* 1811, *communicated by Dr. Villeneuve.*

We learn from Padua that Dr. Quadri, Professor of Anatomy at Bologna, has twice performed an operation for the extraction of cataract without either wounding the transparent cornea or the iris. This method of operating has met with the approbation of the Professors of the University, in presence of whom it was performed. It appears even to be less dangerous than couching. Dr. Quadri will soon publish an account of this method, with the different experiments he has already made upon the subject.—*Journal de Medecine de Corvisart, etc. Nov.* 1810.

*Inflammation of the Spinal Marrow or its Membranes.*

Dr. Bergamaschi, having seen several cases of this disease in the hospital of Padua, communicated the following ones to Dr. Brera, professor of clinical medicine at the University of Parma.

In

In June 1804, a young man, convalescent, undertook a journey on foot, notwithstanding the heat of the season. On his return, worn out with fatigue, he suddenly felt a violent pain in the glutei muscles, opposite the ischiotic notch on the left side; the left leg became affected; the violence of the pains increased, they gradually extended to the sacrum, to the loins, up the whole of the spine, and into the arms; the eyes became prominent, his speech broken, pulse hard and vibrating; the legs and thighs benumbed. This state continued till the ninth day, when the violence of all these symptoms increased; occasional obscure delirium came on, with extreme anxiety, fixed pain along the spine; the muscles of the back were contracted as in a state of tetanus, nor could the patient be raised up without the most excruciating pain; the pulse was very small and irregular, with ischuria and continual perspiration; then came on excessive coldness, difficult respiration, continually increasing, followed by cold viscous sweats, and death. Suppuration was found to have taken place in the whole length of the spinal marrow, the cervical portion of which was least altered; the principal disorder was near the second lumbar vertebra, where it appeared corroded and dissolved.

In the course of the same year, Dr. Bergamaschi saw a young man, who, after a violent effort to raise a heavy weight, felt a painful traction along the spine of the neck down to the back; coldness, with tetanic stiffness of the muscles of the face, neck, and abdomen, came on, followed by difficult respiration; he could not swallow or stretch out his arms without pain, the pulse became frequent, irregular, and the patient died in convulsions. A large quantity of lymph was found between the membranes of the spinal marrow and the sides of the spinal canal; the arteries of the spine were turgid; and the spinal marrow itself of an extraordinary hardness.

A deaf and dumb porter entered the hospital of Padua in March, 1808. He had been ill about a fortnight: his pulse was frequent, hard, and small; his respiration short and painful; the face animated, with a tendency to sleep. Nothing could be learned from him. A synochus gravior was supposed to exist. On the seventh day after his coming to the hospital, delirium came on. He frequently carried his hands to his loins, which, together with the violent outcries he made on moving the body, gave the idea of excessive pain in that part. On the eighth and ninth he refused to swallow any thing, became furious, subsultus tendinum came on, and his limbs were seized with irregular convulsive motions: tenth, he vomited, paralysis of the neck of the bladder now occurred, and a stroke of apoplexy put an end to his existence. It was discovered after death that he had received ceived some violent blows with a stick on the back and loins about the end of February. On examining the spine, a large quantity of water was found between the bone and membranes; a considerable quantity of serum was also poured out between these and the spinal marrow, especially at the lower part, where it formed a sort of tumor. Dr. Bergamaschi concludes, from these and several other cases, that, independent of fever, *spinitis* is characterised by an acute pain along the spine of the vertebræ, and which becomes dreadful on motion; that the nervous symptoms are truly pathognomic, though under various forms of tetanus, irregular convulsions, palsy, or numbness of the limbs; and that, whenever such symptoms are seen to follow any of the causes of spinitis, the disease may be supposed to exist: it is also characterised by pain felt on touching the back, similar to that experienced on touching the abdomen during inflammation of its viscera. Violent efforts, falls, insolation in very hot weather, percussion, softening or swelling of one of the vertebræ or interosseous substance, the action of raising a child by its head, fracture or distraction of the vertebræ, are all considered as causes of this disease, which is seldom induced by any internal ones, unless by retropulsion of some cutaneous affection.—*Bulletin des Sciences Medicales. Paris, Feb.* 1811.

